# Immunological failure of first-line and switch to second-line antiretroviral therapy among HIV-infected persons in Tanzania: analysis of routinely collected national data

**DOI:** 10.1111/tmi.12507

**Published:** 2015-04-02

**Authors:** Fiona M Vanobberghen, Bonita Kilama, Alison Wringe, Angela Ramadhani, Basia Zaba, Donan Mmbando, Jim Todd

**Affiliations:** 1 London School of Hygiene & Tropical MedicineLondon, UK; 2 Mwanza Intervention Trials Unit, National Institute for Medical ResearchMwanza, Tanzania; 3 National AIDS Control ProgramDar es Salaam, Tanzania; 4 Ministry of Health and Social WelfareDar es Salaam, Tanzania; 5 Kilimanjaro Christian Medical University CollegeMoshi, Tanzania

**Keywords:** adult, antiretroviral therapy, CD4 lymphocyte count, risk factors, Tanzania, treatment failure

## Abstract

**Objectives:**

Rates of first-line treatment failure and switches to second-line therapy are key indicators for national HIV programmes. We assessed immunological treatment failure defined by WHO criteria in the Tanzanian national HIV programme.

**Methods:**

We included adults initiating first-line therapy in 2004–2011 with a pre-treatment CD4 count, and ≥6-months of follow-up. We assessed subhazard ratios (SHR) for immunological treatment failure, and subsequent switch to second-line therapy, using competing risks methods to account for deaths.

**Results:**

Of 121 308 adults, 7% experienced immunological treatment failure, and 2% died without observed immunological treatment failure, over a median 1.7 years. The 6-year cumulative probability of immunological treatment failure was 19.0% (95% CI 18.5, 19.7) and of death, 5.1% (4.8, 5.4). Immunological treatment failure predictors included earlier year of treatment initiation (*P *< 0.001), initiation in lower level facilities (SHR = 2.23 [2.03, 2.45] for dispensaries *vs*. hospitals), being male (1.27 [1.19, 1.33]) and initiation at low or high CD4 counts (for example, 1.78 [1.65, 1.92] and 5.33 [4.65, 6.10] for <50 and ≥500 *vs*. 200–349 cells/mm^3^, respectively). Of 7382 participants in the time-to-switch analysis, 6% switched and 5% died before switching. Four years after immunological treatment failure, the cumulative probability of switching was 7.3% (6.6, 8.0) and of death, 6.8% (6.0, 7.6). Those who immunologically failed in dispensaries, health centres and government facilities were least likely to switch.

**Conclusions:**

Immunological treatment failure rates and unmet need for second-line therapy are high in Tanzania; virological monitoring, at least for persons with immunological treatment failure, is required to minimise unnecessary switches to second-line therapy. Lower level government health facilities need more support to reduce treatment failure rates and improve second-line therapy uptake to sustain the benefits of increased coverage.

**Objectifs:**

Les taux d’échec du traitement de 1^ère^ ligne et les passages au traitement de 2^nde^ ligne sont des indicateurs clés pour les programmes nationaux VIH. Nous avons évalué l’échec immunologique du traitement selon les critères de l’OMS dans le programme national VIH tanzanien.

**Méthodes:**

Nous avons inclus les adultes entreprenant une thérapie de 1^ère^ ligne entre 2004 et 2011 avec une numération des CD4 prétraitement disponible et un suivi ≥6 mois. Nous avons évalué les rapports en dessous du risque pour l’échec immunologique du traitement et le passage subséquent à la thérapie de 2^nde^ ligne, en utilisant les méthodes de risques concurrents pour tenir compte des décès.

**Résultats:**

Sur 121.308 adultes, 7% ont connu un échec immunologique du traitement et 2% sont décédés sans observation d’échec immunologique du traitement, sur une médiane de 1,7 ans. La probabilité cumulée d’échec immunologique du traitement sur six ans était de 19,0% (IC95%: 18,5 à 19,7) et 5,1% (4,8 à 5,4) de décès. Les prédicteurs d’échecs immunologiques du traitement comprenaient: l'instauration précoce du traitement (p <0,001), l'initiation dans les établissements de niveau inférieur (SHR = 2,23 [2,03 à 2,45] pour les dispensaires versus les hôpitaux), le sexe masculin (1,27 [1,19 à 1,33]) et l'initiation du traitement à des taux de CD4 faibles ou élevés (par exemple, 1,78 [1,65 à 1,92] et 5,33 [4,65 à 6,10] pour des taux <50 et ≥500 versus des taux compris entre 200 et 349 cellules/mm^3^, respectivement). Sur 7.382 participants à l'analyse sur le moment du changement de traitement, 6% ont changé de traitement et 5% sont décédés avant le changement. Quatre ans après l’échec immunologique du traitement, la probabilité cumulative du changement de traitement était de 7,3% (6,6 à 8,0) et de 6,8% (6,0 à 7,6) pour les décès. Ceux qui ont connu un échec immunologique dans les dispensaires, les centres de santé et les établissements gouvernementaux étaient les moins susceptibles de changer de traitement.

**Conclusions:**

Les taux d’échecs immunologiques du traitement et les besoins non satisfaits pour le traitement de 2^nde^ ligne sont élevés en Tanzanie. La surveillance virologique, au moins pour les personnes avec un échec immunologique de traitement, est nécessaire pour minimiser les passages inutiles au traitement de 2^nde^ ligne. Les établissements de santé gouvernementaux de niveau inférieur ont besoin de plus de support pour réduire les taux d’échec de traitement et pour améliorer l'adoption de la thérapie de 2^nde^ ligne afin de maintenir les avantages d'une couverture accrue.

**Objetivos:**

Las tasas de fallo de la terapia de primera línea y los cambios a la terapia de segunda línea son indicadores claves para los programas nacionales de VIH. Hemos evaluado los fallos en el tratamiento inmunológico definidos según criterios de la OMS dentro del programa nacional para VIH en Tanzania.

**Métodos:**

Hemos incluido adultos que iniciaban la terapia de primera línea entre el 2004-2011 con un conteo de CD4 antes de recibir el tratamiento, y tras ≥6 meses de seguimiento. Hemos evaluado los subíndices de riesgo del fallo inmunológico en el tratamiento, y el cambio subsecuente a la segunda línea de tratamiento, utilizando análisis de riesgo competitivo para explicar las muertes.

**Resultados:**

De 121,308 adultos, un 7% experimentó fallo inmunológico, y un 2% murió sin observarse un fallo inmunológico en el tratamiento, a lo largo de una mediana de 1.7 años. La probabilidad acumulada a lo largo de seis años de fallo terapéutico inmunológico era del 19.0% (IC 95% 18.5, 19.7) y de muerte del 5.1% (4.8,5.4). Los vaticinadores de fallo terapéutico inmunológico incluían haber empezado el tratamiento un año antes (p<0.001), haberlo iniciado en centros de menor nivel (SHR=2.23 [2.03,2.45] para dispensarios versus hospitales), ser ombre (1.27 [1.19,1.33]) e iniciar con conteos de CD$ bajos o altos (por ejemplo, 1.78 [1.65,1.92] y 5.33 [4.65,6.10] para <50 y ≥500 versus 200-349 células/mm^3^, respectivamente). De 7,382 participantes en el análisis de tiempo-hasta-el-cambio, un 6% cambió y un 5% murió antes del cambio. Cuatro años después del fallo terapéutico inmunológico, la probabilidad acumulativa de cambiar era del 7.3% (6.6,8.0) y de muerte, del 6.8% (6.0,7.6). Aquellos que tuvieron un fallo terapéutico inmunológico en los dispensarios, centros sanitarios y centros gubernamentales tenían una menor probabilidad de cambiar.

**Conclusiones:**

Las tasas de fallo terapéutico inmunológico y una necesidad de segunda línea de tratamiento no resuelta son altas en Tanzania; la monitorización virológica, al menos en el caso de personas con fallo terapéutico inmunológico, es necesaria para minimizar los cambios innecesarios a la segunda línea de tratamiento. Los centros sanitarios gubernamentales de menor nivel requieren de más apoyo para reducir las tasas de fallo terapéutico y mejorar la aceptación de la segunda línea de tratamiento asegurando la continuidad de los beneficios de una mayor cobertura.

## Introduction

The year 2012 saw the largest annual increase of HIV-positive persons receiving antiretroviral therapy (ART), with 9.7 million people in low- and middle-income countries on ART 1. In 21 African countries with the highest HIV burden, two-thirds of people in need of treatment in 2012 were receiving ART 1. Furthermore, with recent treatment guideline changes, the number of people eligible for first-line treatment will increase 2. While much remains to be done to reach all in need of treatment, the focus has shifted to the implications of providing long-term treatment for what, under the right care, has become a chronic condition.

Monitoring persons on ART for treatment failure is essential to ensure that their treatment remains potent and to enable timely switches from first- to second-line therapy. In South Africa, where routine viral load monitoring is performed, the proportion of persons switching 3–5 years after treatment initiation was approximately 10% 3,4, whereas in settings without routine viral load monitoring, such as Malawi and Zambia prior to 2011, switching rates were much lower (approximately 2% by 3 years) 4. Delayed switching increases the risk of drug resistance 5,6 and subsequent higher viral load 7–9 and hence impairs clinical outcomes 2, while early, unnecessary switching may reduce treatment options and increase costs. WHO recommends routine viral load monitoring for persons on ART 2, but this remains too expensive for resource-limited countries such as Tanzania. In the absence of viral load monitoring, treatment failure is diagnosed using immunological and clinical criteria 2, as implemented in Tanzanian policy 10–12. To date, there is a paucity of data on the rates and predictors of first-line treatment failure, and the use of second-line therapy, within national programmes using immunological and/or clinical criteria.

Tanzania had an estimated 1.3 million HIV-infected adults in 2011 13. Of these, approximately 370 000 adults (28%) were enrolled in care, and approximately 260 000 were receiving ART, representing 65% in need of treatment 13. Our aim was to investigate the rate and predictors of immunological treatment failure, and subsequent switch to second-line therapy, among HIV-infected adults receiving therapy through the Tanzania government programme.

## Methods

### HIV care and treatment in Tanzania

The Tanzanian National AIDS Control Programme (NACP) provides HIV prevention, care and treatment services. In late 2003, the first HIV/AIDS Care and Treatment Plan was launched, and free ART was rolled out from 2004. By the end of 2011, >1100 facilities were approved to provide care and treatment services, estimated to enable >1 million persons potentially to access ART 13.

HIV-positive persons enrolling in care and treatment clinics are assessed for ART eligibility, defined pre-2012 (data collection period) as CD4 count <200 cells/mm^3^, or CD4 count <350 cells/mm^3^ and WHO stage III, or WHO stage IV regardless of CD4 count 10,11. Persons not yet eligible for ART are encouraged to attend clinics six-monthly for pre-treatment monitoring, while those on treatment attend monthly. First-line treatment consists of 2 nucleoside/nucleotide-reverse transcriptase inhibitors (NRTIs) and a non-NRTI, while second-line therapy included 2 NRTIs plus a protease inhibitor. Individual paper-based records, including unique, nationally attributed patient identifiers, are maintained at each facility, and subsequently electronically entered by data entry clerks before being regularly submitted to the national database.

### Study population

We included data from clinics reporting electronic, individual-level data to the end of 2011. We included persons who initiated first-line ART in 2004–2011 aged ≥15 years with a pre-ART CD4 count available and who completed ≥6 months of follow-up.

### Definition of immunological treatment failure

The Tanzanian 2005 National Guidelines for the Clinical Management of HIV and AIDS defined immunological treatment failure as CD4 count <30% of peak on-treatment value or <pre-treatment levels 10; this definition was revised in 2009 to CD4 count <50% of peak value within 6 months or <pre-treatment levels 11. This resembles the WHO 2010 Antiretroviral Therapy for HIV Infection in Adults and Adolescents guidelines which defined immunological treatment failure as CD4 count <50% of peak value or <pre-treatment levels, or persistently <100 cells/mm^3^ 14; the WHO guidelines were revised in 2013 to remove the criterion of a 50% drop 2. For this analysis, we used the WHO 2010 guidelines, with a second consecutive confirmatory CD4 count for the definition of immunological treatment failure, to rule out transient drops in CD4 counts due to other infections or measurement error. Immunological treatment failure was only defined ≥6 months after treatment initiation 2. We also considered a less strict definition of immunological treatment failure, which did not require a confirmatory CD4 count (except for the criterion of CD4 count <100 cells/mm^3^, as the WHO guidelines explicitly define immunological treatment failure among individuals with CD4 counts ‘persistently’ <100 cells/mm^3^).

### Statistical methods

We assessed immunological treatment failure and death rates and predictors using competing risks methods to account for deaths. Death is a competing risk for immunological treatment failure because its occurrence prevents us from observing immunological treatment failure. In such situations, standard Cox proportional hazards models are not appropriate, and instead competing risks models are required. Such models yield subhazards ratios which, although statistically speaking are different, may be interpreted in the same way as hazard ratios derived from Cox models 15,16. Among those with immunological treatment failure, we assessed switch to second-line therapy, using similar methods. Loss to follow-up was considered uninformative. Body mass index (BMI) was not included in multivariable models, as it was missing for approximately 70% of visits, mainly due to missing height.

Data were censored at 31 December 2011. If a CD4 count was not recorded for >12 months, then follow-up was censored at 12 months after the last CD4 count, but that person could re-enter the risk set if another CD4 count was subsequently recorded. If the person reappeared with immunological treatment failure, then he/she was considered to have immunologically failed at 12 months after the last CD4 count recorded before the gap. Time-dependent variables at ART initiation or switch were defined as the closest up to 3 months earlier, and if none then up to 2 weeks after (except for CD4 count at treatment initiation, which permitted up to 4 weeks after, to allow for delayed reporting of CD4 counts). We performed a sensitivity analysis using 6 instead of 12 months for censoring follow-up. We performed a second sensitivity analysis including only data from 2009 or later (due to concerns about the changes in ART provision, with more being provided by health centres and dispensaries in later years).

For the analysis of switch to second-line therapy, individuals who changed to an unknown ART regimen were censored at that time; those with missing ART information were considered to still be continuing on their first-line regimen. Intermittent regimens of duration ≤14 days were ignored. Individuals with missing ART information from the date when they were last known to be on first-line therapy until the date they switched to second-line therapy were assumed to have switched at the mid-point between these dates. Participants who changed therapy on the day of immunological treatment failure were given 1 day of follow-up. Analyses were conducted using Stata version 12 (StataCorp. 2011. *Stata Statistical Software: Release 12*. College Station, TX: StataCorp LP). *P*-values are 2 sided.

### Ethical considerations

This analysis was conducted on routinely collected data under the auspices of the NACP and approved by the London School of Hygiene & Tropical Medicine ethics committee. Unique patient identifiers were used to preserve anonymity, and all names and personal identifiers were removed before analysis.

## Results

In 348 clinics, 243 844 adults initiated first-line ART. Of these, 71 285 (29%) participants did not have a pre-treatment CD4 count recorded: 23 038 (32%) were WHO stage IV (among whom treatment should have been initiated regardless of CD4 count as per treatment guidelines 10,11), but 5608 (8%) did not have WHO stage recorded, and 26 599 (37%), 11 180 (16%) and 4860 (7%) were WHO stages I, II and III, respectively (perhaps suggesting missing CD4 count data). Of the remaining 172 559 participants, 11 397 (7%) died within the first 6 months after treatment initiation, 13 625 (8%) initiated treatment in the last 6 months of 2011 and therefore had <6 months of follow-up, and 26 229 (15%) were lost to follow-up within 6 months; these participants are excluded.

Of the remaining 121 308 participants (representing all 348 clinics), 73% initiated ART in hospitals and 67% initiated in government-run facilities (Table1). Two-thirds of participants were female, 55% were married or cohabiting, and 89% were working. A total of 26% of participants initiated ART with low BMI (<18.5 kg/m^2^), 16% with WHO stage IV and 73% with low CD4 count (<200 cells/mm^3^). The most common first-line ART regime was stavudine based (61%), mainly driven by data from earlier years. The use of zidovudine, lamivudine and nevirapine or efavirenz increased from 8% to 10%, respectively, in 2008, to 36% and 40%, respectively, in 2011, following the elimination of stavudine in 2010 (Table S1).

**Table 1:** Participant characteristics at ART initiation and immunological treatment failure

		At ART initiation*		At immunological failure*,†	
		*N* = 121 308	%	*N* = 7382	%
Health facility level	Hospital	87 770	72.7	4712	64.3
	Health centre	16 798	13.9	1189	16.2
	Dispensary	13 131	10.9	1027	14.0
	Other‡	2995	2.5	397	5.4
Health facility type	Government	74 789	66.7	4696	68.8
	Faith-based	28 343	25.3	1712	25.1
	Private	8947	8.0	413	6.1
Year	Up to end 2005	5951	4.9	42	0.6
	2006	12 181	10.0	471	6.4
	2007	19 770	16.3	954	12.9
	2008	26 158	21.6	1396	18.9
	2009	25 726	21.2	1559	21.1
	2010	22 121	18.2	1581	21.4
	2011	9401	7.7	1379	18.7
Sex	Male	40 055	33.0	2630	35.6
	Female	81 250	67.0	4752	64.4
Age, years	15 to 29	23 412	19.3	953	12.9
	30 to 39	50 750	41.8	3063	41.5
	40 to 49	31 848	26.3	2263	30.7
	≥50	15 278	12.6	1099	14.9
Marital status§	Single	24 757	22.2	1648	25.2
	Married or cohabiting	61 586	55.3	3493	53.4
	Divorced or separated	11 866	10.7	635	9.7
	Widowed	13 156	11.8	765	11.7
Functional status	Working	102 301	88.7	6980	96.9
	Ambulatory	11 866	10.3	177	2.5
	Bed-ridden	1177	1.0	49	0.7
Weight, kg	<45	21 754	18.1	690	9.4
	45 to <55	47 019	39.1	2292	31.2
	≥55	51 633	42.9	4365	59.4
BMI·	Underweight	11 035	26.2	427	13.4
	Normal	25 097	59.6	2030	63.7
	Overweight	6002	14.2	732	23.0
WHO stage§	I	11 586	10.4	562	9.0
	II	27 636	24.9	1445	23.0
	III	53 603	48.3	3169	50.5
	IV	18 158	16.4	1102	17.6
CD4 count, cells/mm^3^	<50	24 339	20.1	1822	24.7
	50 to 199	64 753	53.4	3684	49.9
	200 to 349	27 375	22.6	1333	18.1
	350 to 499	3250	2.7	383	5.2
	≥500	1591	1.3	158	2.1
First ART regimen§	Stavudine-based	73 402	60.5	5287	71.6
	Zidovudine-based	46 739	38.5	2008	27.2
	Other first line	1167	1.0	87	1.2
Time on first-line ART, years	<1			2950	40.0
	1 to <2			2475	33.5
	≥2			1957	26.5

ART, antiretroviral therapy; BMI, body mass index.

* Values are number (% of those with non-missing data).

† Restricted to those included in the switching analysis (see main text).

‡ ‘Other’ facilities predominantly included institutional facilities with restricted access.

§ At ART initiation (not updated at immunological failure; marital status only recorded at enrolment into care).

¶ BMI categorised as underweight (<18.5 kg/m^2^), normal (18.5 to <25.0 kg/m^2^) or overweight (≥25 kg/m^2^).

Nearly two-thirds of participants (65%) did not have any gaps in their follow-up due to CD4 counts not being recorded for >12 months; 28%, 6%, <1% and <1% of participants had one, two, three or four such gaps in their follow-up, respectively. Across all gaps, the median gap length was 7 months, with an interquartile range of 3–13 months.

### Immunological failure

**Figure 1:**
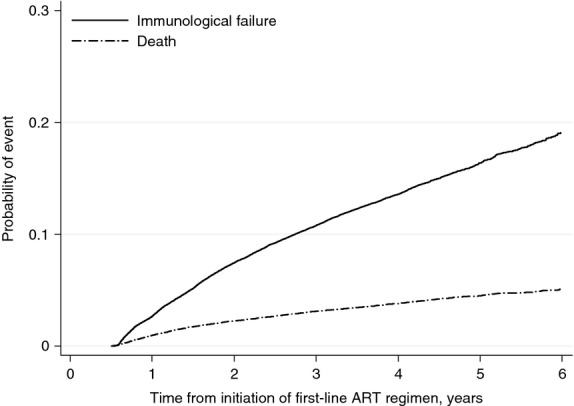
Probability of immunological treatment failure or death, following initiation of first-line ART. ART, antiretroviral therapy. *Y*-axis truncated at 0.3. Persons with <6 months of follow-up (including due to death) were excluded from the analyses. Immunological failure was not defined until at least 6 months after treatment initiation.

Subsequent to the first 6 months on ART, 8384 (7%) participants experienced immunological treatment failure and 2486 (2%) died without immunological treatment failure being observed, over a median of 1.7 years (maximum 8 years). Of those experiencing immunological treatment failure, 1995 (24%) participants had CD4 counts <pre-treatment levels, 1400 (17%) <50% of on-treatment peak, 2625 (31%) <100 cells/mm^3^, and 2364 (28%) had a combination of these components (Table S2). The cumulative probability of immunological treatment failure by 6 years (to when we had sufficient data for reliable estimation) was 19.0% (95% CI: 18.5, 19.7) and of death (without immunological treatment failure) was 5.1% (4.8, 5.4; Figure1).

Under the less strict immunological treatment failure definition, 19 380 (16.0%) participants would have been considered to have experienced immunological treatment failure, with cumulative probability of 23.8% (23.5, 24.2) by 3 years and 40.6% (39.8, 41.5) by 6 years.

### Predictors of immunological treatment failure

Using the definition of immunological treatment failure with confirmatory CD4 count, in adjusted analyses, higher risk of immunological treatment failure was found among those who initiated treatment in lower level facilities and in ‘other’ facilities, which predominantly included institutional facilities with restricted access (*P* < 0.001; Table2). However, those in ‘other’ facilities had lowest death rate (0.6 *vs*. 1.1/100 person-years in hospitals). The immunological treatment failure risk was lower in private *vs*. government facilities (subhazard ratio, SHR = 0.59 [95% confidence interval, CI: 0.50, 0.69]), with no difference for faith-based facilities (SHR = 1.01 [0.95, 1.07]). There was lower immunological treatment failure risk with later year of treatment initiation (*P* < 0.001), and death rates decreased from 1.2/100 to 0.5/100 person-years among those who initiated treatment pre-2006 and in 2011, respectively. Females had lower immunological treatment failure risk than men (SHR = 0.79 [0.75, 0.84]). Compared to persons who were married or cohabiting at treatment initiation, single persons were at higher immunological treatment failure risk (SHR = 1.12 [1.05, 1.20]), but there was no evidence of a difference for those divorced or separated, or widowed.

**Table 2:** Associations of participant characteristics at ART initiation with immunological treatment failure and death, after first-line treatment initiation

	Immunological failure			Death (before immunological failure)			Subhazard ratio (95% confidence interval) for immunological failure			
	Rate per 100 person-years	*N* events	Person-years	Rate per 100 person-years	*N* events	Person-years	Univariable models		Full multivariable model	
Health facility level							*P *<* *0.001		*P *<* *0.001	
Hospital	3.2	5776	179 186	1.1	1911	179 186	1		1	
Health centre	3.3	966	28 865	1.0	302	28 865	1.05	0.98, 1.12	1.19	1.10, 1.29
Dispensary	4.8	1133	23 735	0.9	214	23 735	1.51	1.41, 1.61	2.23	2.03, 2.45
Other*	5.8	448	7763	0.6	50	7763	1.75	1.59, 1.93	1.73	1.54, 1.95
Health facility type							*P *=* *0.01		*P *<* *0.001	
Government	3.5	5169	147 962	1.0	1455	147 962	1		1	
Faith-based	3.3	1996	59 877	1.2	714	59 877	0.94	0.89, 0.99	1.01	0.95, 1.07
Private	3.1	540	17 209	0.8	145	17 209	0.91	0.83, 0.99	0.59	0.50, 0.69
Year							*P *<* *0.001		*P *<* *0.001	
Up to end 2005	5.2	1029	19 724	1.2	228	19 724	2.06	1.90, 2.24	2.47	2.22, 2.73
2006	4.8	1732	35 781	1.2	421	35 781	1.86	1.74, 1.99	1.90	1.75, 2.07
2007	3.9	1986	50 880	1.1	553	50 880	1.42	1.33, 1.52	1.41	1.31, 1.52
2008	2.8	1610	56 696	1.1	597	56 696	1		1	
2009	3.2	1376	43 500	1.0	423	43 500	1.1	1.02, 1.18	0.88	0.81, 0.97
2010	2.3	612	27 165	0.8	230	27 165	0.84	0.76, 0.92	0.60	0.54, 0.68
2011	0.6	39	6875	0.5	34	6875	0.39	0.28, 0.54	0.28	0.20, 0.40
Sex							*P *<* *0.001		*P *<* *0.001	
Male	4	3017	76 151	1.4	1065	76 151	1		1	
Female	3.3	5367	164 463	0.9	1421	164 463	0.82	0.79, 0.86	0.79	0.75, 0.84
Age, years							*P *=* *0.60		*P *=* *0.58	
15 to 29	3.4	1537	44 600	0.9	411	44 600	0.98	0.93, 1.04	0.95	0.89, 1.03
30 to 39	3.5	3587	101 822	0.9	932	101 822	1		1	
40 to 49	3.5	2254	64 512	1.0	669	64 512	0.99	0.94, 1.04	1.01	0.94, 1.07
≥50	3.4	1002	29 648	1.6	474	29 648	0.95	0.89, 1.02	0.98	0.90, 1.07
Marital status							*P *<* *0.001		*P *=* *0.004	
Single	3.8	1888	49 109	1.1	517	49 109	1.14	1.08, 1.21	1.12	1.05, 1.20
Married or cohabiting	3.3	3955	118 396	1.0	1155	118 396	1		1	
Divorced or separated	3.2	736	22 679	1.1	239	22 679	0.97	0.90, 1.05	1.06	0.97, 1.16
Widowed	3.2	860	26 536	0.9	252	26 536	0.97	0.90, 1.04	1.05	0.96, 1.14
Functional status							*P *=* *0.83		*P *=* *0.21	
Working	3.4	6774	196 437	0.9	1852	196 437	1		1	
Ambulatory	3.4	863	25 060	1.6	394	25 060	0.98	0.91, 1.05	0.92	0.85, 1.01
Bed-ridden	3.4	80	2335	1.9	45	2335	0.97	0.78, 1.21	0.99	0.78, 1.25
Weight, kg							*P *<* *0.001		*P *=* *0.03	
<45	3.8	1600	42 615	1.4	615	42 615	1.14	1.08, 1.21	1.07	0.99, 1.16
45 to <55	3.6	3258	91 619	1.0	961	91 619	1.09	1.04, 1.14	1.08	1.02, 1.14
≥55	3.3	3430	104 635	0.8	887	104 635	1		1	
BMI†							*P *=* *0.001			
Underweight	4.2	1022	24 193	0.9	227	24 193	1.12	1.04, 1.20		
Normal	3.8	2095	55 456	0.6	338	55 456	1			
Overweight	3.5	474	13 516	0.5	63	13 516	0.93	0.84, 1.03		
WHO stage							*P *<* *0.001		*P *=* *0.03	
I	2.8	638	22 513	0.5	119	22 513	0.83	0.76, 0.90	0.92	0.84, 1.01
II	3.2	1630	51 678	0.8	431	51 678	0.93	0.87, 0.98	1.04	0.98, 1.11
III	3.4	3589	104577	1.1	1122	104 577	1		1	
IV	3.7	1288	34 879	1.5	529	34 879	1.07	1.01, 1.14	0.94	0.88, 1.02
CD4 count, cells/mm^3^							*P *<* *0.001		*P *<* *0.001	
<50	5.8	2741	47 534	1.4	649	47 534	1.95	1.83, 2.08	1.78	1.65, 1.92
50 to 199	2.5	3402	133 708	1.0	1330	133 708	0.86	0.80, 0.91	0.78	0.72, 0.84
200 to 349	2.9	1496	51 208	0.8	411	51 208	1		1	
350 to 499	6.8	382	5623	1.0	58	5623	2.36	2.11, 2.64	2.51	2.20, 2.86
≥500	14.2	363	2548	1.5	38	2548	4.96	4.44, 5.55	5.33	4.65, 6.10
First ART regimen							*P *<* *0.001		*P *<* *0.001	
Stavudine-based	3.6	6059	167 123	1.1	1840	167 123	1		1	
Zidovudine-based	3.1	2233	72 418	0.9	637	72 418	0.9	0.85, 0.94	1.14	1.68, 1.21
Other first line	8.5	92	1080	0.8	9	1080	3.36	2.73, 4.13	6.12	4.90, 7.65

ART, antiretroviral therapy; BMI, body mass index.

‘1’ indicates the reference category.

* ‘Other’ facilities predominantly included institutional facilities with restricted access.

† BMI categorised as underweight (<18.5 kg/m^2^), normal (18.5 to <25.0 kg/m^2^) or overweight (≥25 kg/m^2^).

**Table 3:** Rates and predictors of switching, after immunological treatment failure

				Subhazard ratio (95% confidence interval)			
	Rate per 100 person-years	*N* events	Person-years	Univariable models		Full multivariable model	
Health facility level				*P *<* *0.001		*P *<* *0.001	
Hospital	2.7	315	11 462	1		1	
Health centre	1.3	27	2075	0.42	0.29, 0.63	0.43	0.26, 0.71
Dispensary	1.1	21	1964	0.31	0.20, 0.48	0.50	0.27, 0.93
Other*	5.9	53	900	2.02	1.50, 2.71	2.27	1.52, 3.39
Health facility type				*P *<* *0.001		*P *<* *0.001	
Government	2.1	226	10 917	1		1	
Faith-based	4.8	176	3667	2.26	1.86, 2.75	2.29	1.79, 2.91
Private	1.2	10	825	0.53	0.28, 0.99	†	†
Year				*P *=* *0.004		*P *<* *0.001	
Up to end 2005	3.6	8	223	1.70	0.83, 3.49	1.08	0.35, 3.32
2006	1.8	39	2211	0.87	0.61, 1.26	1.21	0.76, 1.90
2007	2.0	75	3676	0.90	0.67, 1.21	1.25	0.88, 1.77
2008	2.7	111	4184	1		1	
2009	3.0	100	3339	0.90	0.69, 1.19	0.86	0.62, 1.19
2010	2.4	52	2186	0.55	0.39, 0.76	0.47	0.31, 0.70
2011	4.7	31	666	0.65	0.43, 0.97	0.41	0.25, 0.65
Sex				*P *=* *0.005		*P *=* *0.03	
Male	3.1	174	5668	1		1	
Female	2.2	242	10 818	0.76	0.62, 0.92	0.77	0.60, 0.97
Age, years				*P *=* *0.23		*P *=* *0.76	
15 to 29	3.0	67	2253	1.32	1.00, 1.76	1.07	0.75, 1.52
30 to 39	2.3	160	7003	1		1	
40 to 49	2.5	123	4984	1.06	0.84, 1.34	0.94	0.72, 1.23
≥50	2.9	65	2231	1.19	0.89, 1.58	0.86	0.61, 1.23
Marital status‡				*P *=* *0.21		*P *=* *0.35	
Single	2.9	102	3483	1.20	0.94, 1.53	1.21	0.93, 1.59
Married or cohabiting	2.4	180	7442	1		1	
Divorced or separated	2.0	27	1349	0.83	0.55, 1.25	0.92	0.60, 1.42
Widowed	2.8	48	1698	1.20	0.87, 1.65	1.24	0.87, 1.75
Functional status				*P *=* *0.43		*P *=* *0.34	
Working	2.6	394	15 126	1		1	
Ambulatory	1.6	7	439	0.64	0.30, 1.35	0.50	0.20, 1.26
Bed-ridden	1.6	2	123	0.67	0.16, 2.74	0.94	0.22, 4.08
Weight, kg				*P *=* *0.92		*P *=* *0.54	
<45	2.7	39	1446	0.99	0.71, 1.39	1.05	0.70, 1.59
45 to <55	2.5	124	4939	0.96	0.77, 1.19	0.87	0.67, 1.14
≥55	2.5	253	10 058	1		1	
WHO stage‡				*P *<* *0.001		*P *<* *0.001	
I	4.2	51	1201	1.73	1.27, 2.37	1.64	1.18, 2.28
II	3.0	86	2867	1.16	0.89, 1.50	1.11	0.84, 1.47
III	2.5	171	6882	1		1	
IV	1.6	37	2288	0.63	0.44, 0.89	0.56	0.38, 0.81
CD4 count, cells/mm^3^				*P *<* *0.001		*P *<* *0.001	
<50	2.9	115	3975	2.16	1.51, 3.11	6.33	4.03, 9.95
50 to 199	3.0	255	8543	2.31	1.65, 3.23	3.70	2.42, 5.67
200 to 349	1.3	39	2927	1		1	
≥350	0.7	7	1041	0.48	0.21, 1.06	0.52	0.20, 1.36
First ART regimen‡				*P *=* *0.07		*P *<* *0.001	
Stavudine-based	2.3	301	13 018	1		1	
Zidovudine-based	3.4	115	3414	1.22	0.99, 1.52	1.76	1.36, 2.29
Other first line	0	0	55	§		§	
Time on first-line ART, years				*P *<* *0.001		*P *<* *0.001	
<1	1.2	101	8378	1		1	
1 to <2	2.9	155	5433	2.12	1.65, 2.72	2.34	1.72, 3.17
≥2	6.0	160	2674	3.58	2.80, 4.58	5.34	3.84, 7.44

ART, antiretroviral therapy.

‘1’ indicates the reference category.

* ‘Other’ facilities predominantly included institutional facilities with restricted access.

† Not reliably estimable as few switches to second-line therapy, therefore omitted this category from the model.

‡ At ART initiation rather than immunological failure (marital status only recorded at CTC enrolment).

§ Omitted from the model as no one in this category was observed to switch to second-line therapy.

Persons initiating treatment with lower weight were at somewhat higher risk of immunological treatment failure (SHR = 1.07 [0.99, 1.16] and 1.08 [1.02, 1.14] for <45 and 45 to < 55 *vs*. ≥55 kg, respectively). There was some difference in immunological treatment failure risk by WHO stage at treatment initiation (*P* = 0.03), although no clear trend across the stages. Of note, the competing risk of death varied by stage (0.5 *vs*. 1.5/100 person-years for WHO stage I and IV, respectively). Persons who initiated with the lowest CD4 counts were at higher risk of immunological treatment failure (SHR = 1.78 [1.65, 1.92] for <50 *vs*. 200–349 cells/mm^3^). However, persons initiating with high CD4 counts were also at higher immunological treatment failure risk (SHR = 2.51 [2.20, 2.86] and 5.33 [4.65, 6.10] for 350–499 and ≥500 *vs*. 200–349 cells/mm^3^, respectively). In the unadjusted model, persons who initiated on zidovudine-based regimens had a lower immunological treatment failure risk *vs*. stavudine-based regimens; this relationship was reversed once we adjusted for confounders (SHR = 1.14 [1.06, 1.21]). Persons who initiated treatment with other regimens had much higher immunological treatment failure risk (SHR = 6.12 [4.90, 7.65] *vs*. stavudine based). There was no evidence of a difference in immunological treatment failure risk by age (*P* = 0.58) or functional status (*P* = 0.21). Variable selection to obtain a parsimonious model (removing variables in a stepwise fashion with *P* > 0.05) yielded similar results to the full model. Sensitivity analyses censoring follow-up after 6 rather than 12 months, or including only participants who initiated in 2009 or later, yielded broadly similar results.

### Switch to second-line therapy

Of 8384 persons who immunologically failed on first-line therapy, 135 (2%) had previously used second-line therapy, 276 (3%) had previously taken an unknown regimen, and 591 (7%) had an immunological treatment failure date estimated at 12 months after the last CD4 count before a gap of >12 months; these persons are excluded from the following analyses. Of the remaining 7382 (88%) participants, 40% had been on first-line ART for <1 year, 34% for 1 to <2 years and 27% for ≥2 years (Table1). The distribution of participant characteristics at the time of immunological treatment failure broadly reflected those at ART initiation. The proportions of participants with CD4 counts of <50, 50–199, 200–349, 350–499 and ≥500 cells/mm^3^ at immunological treatment failure were 25%, 50%, 18%, 5% and 2%, respectively.

**Figure 2:**
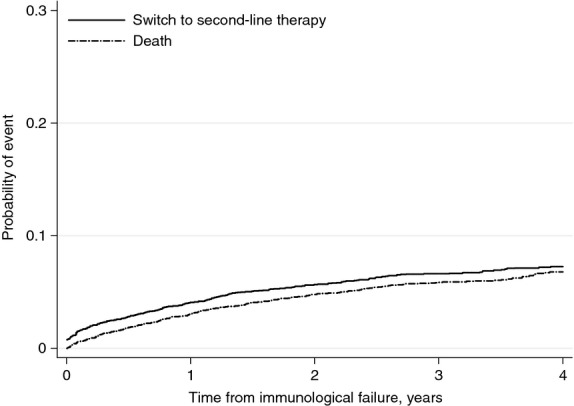
Probability of switch from first- to second-line ART or death, following immunological treatment failure. ART, antiretroviral therapy. *Y*-axis truncated at 0.3. Participants who changed therapy on the day of immunological failure were given 1 day of follow-up, so that they were included in the time-to-event analyses.

Overall, 416 (6%) persons were observed to subsequently switch to second-line therapy, while 355 (5%) died before switching. By 4 years after immunological treatment failure, the cumulative probability of switching was 7.3% (95% CI: 6.6, 8.0) and of death 6.8% (6.0, 7.6; Figure2).

The most common second-line regimen to which people switched was abacavir, didanosine and ritonavir-boosted lopinavir (*n* = 343; 82%), followed by tenofovir, emtricitabine and ritonavir-boosted lopinavir (43; 10%). The reasons for switch were not reported for 162 (39%) individuals; of those given, the most common reasons were immunological treatment failure (184; 72%) or clinical treatment failure (20; 8%).

### Predictors of switch to second-line therapy

In adjusted analyses, there were large differences in the switching rates by facility level and type, with those who immunologically failed in health centres and dispensaries being less likely to switch than those in hospitals (SHR = 0.43 [95% CI: 0.26, 0.71] and 0.50 [0.27, 0.93], respectively), and those in ‘other’ facilities more likely to switch (SHR = 2.27 [1.52, 3.39]). People who experienced immunological treatment failure in faith-based facilities were much more likely to switch than those in government facilities (SHR = 2.29 [1.79, 2.91]). We observed less frequent switching with later year of immunological treatment failure (*P* < 0.001). Women were less likely to switch than men (SHR = 0.77 [0.60, 0.97]). Persons at lower WHO stage at treatment initiation were more likely to switch (*P* < 0.001; for example, SHR = 1.64 [1.18, 2.28] for WHO stage I *vs*. III). Persons with lower CD4 count at immunological treatment failure were much more likely to switch (*P *<* *0.001; for example, SHR = 6.33 [4.03, 9.95] for <50 *vs*. 200–349 cells/mm^3^). Persons who had initiated ART on zidovudine-based therapy were more likely to switch than those on stavudine-based regimens (SHR = 1.76 [1.36, 2.29]). There was increasing probability of switch with increasing time on therapy (*P *<* *0.001). There was no evidence of a difference in switching rates by age (*P *=* *0.76), marital status (*P *=* *0.35), functional status (*P *=* *0.34) or weight (*P *=* *0.54).

## Discussion

In this study of >120 000 HIV-infected adults initiating first-line therapy in Tanzania, the need for second-line therapy was high, with immunological treatment failure rates of 19% by 6 years after treatment initiation. The analysis was restricted to persons with ≥6 months of follow-up, excluding the 7% of people who died within 6 months; nonetheless, over the following 6 years, there was a 5% cumulative probability of death without observed immunological treatment failure. After immunological treatment failure, the cumulative probability over 4 years of switching to second-line therapy was 7%, which was approximately the same as that of death (7%).

To our knowledge, this is the first study to assess immunological treatment failure rates and switches to second-line therapy among adults on first-line ART using national routinely collected data. In a recent study from Nigeria, which used the same WHO criteria for immunological treatment failure but without a confirmatory CD4 count, the cumulative probability of immunological treatment failure was approximately 35% by 3 years, similar to our estimation of 24% under the less strict immunological treatment failure definition 17. When a confirmatory CD4 count was incorporated in the Nigerian analysis, the overall proportion of participants experiencing immunological treatment failure reduced from 32% to 10% and therefore the cumulative immunological treatment failure probability when incorporating a confirmatory CD4 count (not directly reported) is likely to be similar to that observed under the main immunological treatment failure definition in our study. The differences in the estimated immunological treatment failure rates between definitions requiring and not requiring a confirmatory CD4 count are large. CD4 count measurement is known to have large variability and CD4 count trajectories may display transient changes; thus, we believe that it is unlikely that the immunological treatment failure rates are as high as suggested by the unconfirmed criteria, hence reinforcing the importance of a confirmatory CD4 count, which is typically what clinicians seek in practice.

Encouragingly, immunological treatment failure rates dropped with later calendar year of ART initiation, with 72% lower risk among those who initiated in 2011 *vs*. 2008, which may be attributable to improvements in care and drug efficacy. Switching rates also decreased over time, with 59% lower ‘risk’ of switching among those who immunologically failed in 2011 *vs*. 2008, perhaps suggesting that the national programme in Tanzania has not yet organised itself for widespread second-line therapy use. The overall low switching rates observed in this study indicate that there is a large unmet need for second-line therapy, and so this should be a future priority for the ART programme if excess morbidity and mortality among persons on ART are to be minimised. Our results likely reflect what clinicians are doing in practice, regardless of national policies, due to barriers in accessing second-line therapy such as lack of availability and higher cost. Approaches to increase coverage to ART, such as decentralisation, could be harnessed to increase access to second-line therapy.

We found important differences in the rates of both immunological treatment failure and switching by the types of facilities participants were attending. The Tanzanian HIV programme has successfully devolved care to lower level clinics, and there are calls for similar initiatives for the management of other chronic diseases 18. However, the higher immunological treatment failure rates and lower switching rates in lower level and particularly government-owned facilities highlight that adequate training and support is required for front-line healthcare workers, along with a stable drug supply chain and adequate equipment, to ensure that consistent services are provided.

We identified key subgroups of the population who may be at higher immunological treatment failure risk including men, single persons, and those with lower weight at ART initiation. Men typically have poorer healthcare-seeking behaviours than women, as illustrated by mean lower CD4 counts at enrolment to HIV care 5,13, poorer ART uptake 19, and the higher immunological treatment failure risk observed in this study. In contrast, we found that women were less likely to switch to second-line therapy than men; the reasons for this are unclear and this finding warrants further investigation. The drivers behind the higher immunological treatment failure risk with zidovudine-based and other first-line regimens, compared to stavudine-based therapy, are unclear. Stavudine has been phased out since 2010, and tenofovir-based regimens are now recommended. Although only a small percentage of participants initiated tenofovir in this cohort, its use is increasing. Both low and high CD4 counts at ART initiation were associated with higher immunological treatment failure risk. Participants starting treatment with CD4 counts <100 cells/mm^3^ would have met the definition for immunological treatment failure if they had two subsequent CD4 counts <100 cells/mm^3^, even if higher than their baseline value. Individuals initiating treatment at high CD4 counts were likely to be different in some way; for example, they may be presenting for care due to an opportunistic infection. While we have controlled for the confounders routinely captured in the national data, such as WHO stage, there may remain residual confounding.

Lower CD4 count at immunological treatment failure was strongly associated with switching; nonetheless, our results indicate that there remains a large need for second-line therapy which is not being met, with the probability of switch among those who have immunologically failed being only 7% by 4 years. The poor predictive ability of immunological treatment failure for virological failure is well known 17,20–24, meaning that persons with a low CD4 count may not necessarily have virologically failed. However, in a setting without routine or targeted viral load monitoring, switching decisions must be made based on the immunological evidence 2, and this is the situation in many countries across sub-Saharan Africa. New and cheaper viral load tests, using dried blood spots, would ideally be used to perform targeted monitoring of persons with immunological treatment failure to minimise unnecessary switches to second-line treatment, as recommended by the WHO 25. Switching persons who have immunological treatment failure, but not virological failure has individual and economic implications, and such persons would be unlikely to benefit from second-line therapy, and therefore, it would be important to assess viral load before switch.

A strength of this study is the use of appropriate statistical methods, namely competing risks analysis, to take into account the correlation between death and immunological treatment failure. A naïve approach would be to use proportional hazards regression, ignoring the competing risk of death for immunological treatment failure. Such an approach underestimates the immunological treatment failure rate, due to deaths occurring in those with unobserved immunological treatment failure. This underestimation may be greater in a resource-poor setting with less-intensive CD4 monitoring. In addition, our results were robust to sensitivity analyses.

While we included over 120 000 persons in this analysis, the 348 clinics included do not represent every region in Tanzania, as the analysis was restricted to clinics who submitted electronic data in 2011. Due to the definition of immunological treatment failure, we were not able to include nearly a third of registered participants as they did not have a baseline CD4 count; it is difficult to know whether this selection has led to bias in our results. Attrition rates from care and treatment clinics in Tanzania are high 26, and it is likely that many deaths remain unreported; therefore, our mortality rates will be underestimates. While we attempted to address incompleteness of immunological data by censoring follow-up when no CD4 count had been recorded for >12 months, it may be that incomplete data contribute to the deaths without immunological treatment failure. Information on causes of death might help inform this question further, but these data are not currently captured. We used the WHO 2010 immunological treatment failure criteria, covering the majority of the data collection period 14; application of the WHO 2013 guidelines would yield lower immunological treatment failure rates 2. The implications of different definitions could be explored, including the incorporation of persons who initiated at WHO stage IV without CD4 measurements recorded. Further, interpretations of immunological treatment failure were required for analysis, for example related to ‘persistent’ CD4 count <100 cells/mm^3^. This raises questions about how the guidelines are interpreted in clinical practice. The guidelines state that transient drops in CD4 count should be ignored, and we attempted to address this by requiring a confirmatory CD4 count for immunological treatment failure, but we may therefore have underestimated the immunological treatment failure rate. However, the immunological treatment failure rates indicated by our less strict definition, which did not require a confirmatory CD4 count, were implausibly high. Detailed information on clinical treatment failure was not captured, although the number of persons switching to second-line therapy in the absence of immunological treatment failure was low, suggesting perhaps that clinical failure – which may be more complex to diagnose – may not be adequately assessed in clinics. This study does not attempt to address the optimal time-to-switch to second-line therapy to minimise adverse outcomes, which is of importance and should be considered for future work. As second-line therapy use increases, work should address outcomes after switch, particularly as a substantial proportion of persons may be expected not to achieve virological suppression 7,27.

In summary, we used national routinely collected data to investigate immunological treatment failure rates in Tanzania; such rates are high, and the need for second-line treatment is not being met. The Tanzanian national control programme has successfully focused on ART roll-out, and this remains crucial, particularly with new WHO guidelines recommending earlier initiation 2. To sustain the benefits of increased coverage, there is a priority to address the need for second-line therapy, and (targeted) virological monitoring is required to minimise unnecessary switches to second-line therapy.

## Supporting Information

Table S1 First-line ART regimen by year of treatment initiation. **Table S2.** Immunological criteria met, for the 8,384 persons who were observed to have immunological treatment failure, by CD4 count at treatment initiation.
