# Supplementary material for: Immunological failure of first-line and switch to second-line antiretroviral therapy among HIV-infected persons in Tanzania: analysis of routinely collected national data
Source: Trop Med Int Health. 2015 Apr 2;20(7):880–92. doi: 10.1111/tmi.12507 (PMC4672690; doi:10.1111/tmi.12507)
Supplement: Table S1 — First-line ART regimen by year of treatment initiation. Table S2. Immunological criteria met, for the 8,384 persons who were observed to have immunological treatment failure, by CD4 count at treatment initiation. [file tmi0020-0880-sd1.docx]

**Supplementary Table 1.** First-line ART regimen by year of treatment initiation

|  | **Up to end 2005** | **2006** | **2007** | **2008** | **2009** | **2010** | **2011** |
| --- | --- | --- | --- | --- | --- | --- | --- |
|  | N=5,951 | N=12,181 | N=19,770 | N=26,158 | N=25,726 | N=22,121 | N=9,401 |
| Stavudine, lamivudine, nevirapine | 5,081 (85%) | 10,251 (84%) | 15,422 (78%) | 20,290 (78%) | 11,938 (46%) | 4,411 (20%) | 1,497 (16%) |
| Stavudine, lamivudine, efavirenz | 191 (3%) | 712 (6%) | 1,122 (6%) | 1,162 (4%) | 834 (3%) | 382 (2%) | 109 (1%) |
| Zidovudine, lamivudine, nevirapine | 428 (7%) | 547 (4%) | 1,190 (6%) | 2,149 (8%) | 4,104 (16%) | 6,382 (29%) | 3,351 (36%) |
| Zidovudine, lamivudine, efavirenz | 251 (4%) | 671 (6%) | 2,035 (10%) | 2,555 (10%) | 8,767 (34%) | 10,533 (48%) | 3,776 (40%) |
| Tenofovir-based first line | 0 (0%) | 0 (0%) | 1 (0%) | 2 (0%) | 80 (0%) | 401 (2%) | 616 (7%) |
| Other first line | 0 (0%) | 0 (0%) | 0 (0%) | 0 (0%) | 3 (0%) | 12 (0%) | 52 (1%) |

ART=antiretroviral therapy.

**Supplementary Table 2.** Immunological criteria met, for the 8,384 persons who were observed to have immunological treatment failure, by CD4 count at treatment initiation

| **CD4 count at treatment initiation, cells/mm^3^** | **Criterion** | | | | **Total** |
| --- | --- | --- | --- | --- | --- |
|  | **CD4 count <CD4 count at treatment initiation** | **CD4 count <50% of on-treatment peak CD4 count** | **CD4 count <100 cells/mm^3^** | **Combination of criteria** |  |
| <50 | 0 (0%) | 362 (13%) | 1,792 (65%) | 587 (21%) | 2,741 (100%) |
| 50-199 | 583 (17%) | 854 (25%) | 833 (24%) | 1,132 (33%) | 3,402 (100%) |
| 200-349 | 931 (62%) | 173 (12%) | 0 (0%) | 392 (26%) | 1,496 (100%) |
| 349-499 | 263 (69%) | 10 (3%) | 0 (0%) | 109 (29%) | 382 (100%) |
| ≥500 | 218 (60%) | 1 (<1%) | 0 (0%) | 144 (40%) | 363 (100%) |
| Total | 1,995 (24%) | 1,400 (17%) | 2,625 (31%) | 2,364 (28%) | 8,384 (100%) |

Some cells are zero by design. For example, if a person initiated treatment with a CD4 count <100 cells/mm^3^ and subsequently had two consecutive CD4 counts below the value at treatment initiation, then that person would be considered as having immunologically-failed with CD4 count both (a) below the CD4 count at treatment initiation and (b) <100 cells/mm^3^, i.e. that person could not fall solely into the category of <CD4 count at treatment initiation.
